# P29 A rare case of complex multi-system treatment resistant rheumatoid arthritis

**DOI:** 10.1093/rap/rkad070.050

**Published:** 2023-09-27

**Authors:** Preethu Anand, Arjun Raj, Veena Patel

**Affiliations:** University Hospitals of Leicester, Leicester, United Kingdom; University Hospitals of Leicester, Leicester, United Kingdom; University Hospitals of Leicester, Leicester, United Kingdom

## Abstract

**Introduction:**

Rheumatoid arthritis (RA) is a chronic progressive systemic inflammatory disorder with multi-organ involvement. The introduction of biologics has revolutionised the disease management. However, we still have 5-20 percentage of patients who pose clinical challenge with treatment resistant and multi-organ involvement. Cervical spine involvement among RA patients is well noted with a prevalence of 24 percent, however involvement of thoracic and lumbar spine is not commonly known. We present one such case which is treatment resistant, multiple organ involvement disease, associated with the axial involvement, complicated by infection posing clinical challenge for management.

**Case description:**

58-year-old Chinese origin woman, initially diagnosed with RA and Sjogren’s syndrome in 2018. RF and Anti-CCP positive with active synovitis of her hands confirmed on ultrasound.

Initially treated with hydroxychloroquine, sulfasalazine, and methotrexate of which she failed to tolerate hydroxychloroquine and sulfasalazine. A year later she developed progressive SOB. Pulmonary function test and CT-thorax confirmed RA related progressive interstitial lung disease. The respiratory team offered anti-fibrotic therapy as a part of clinical trial but she opted for conservative management.

Given the high disease activity score, she had 2 cycles of rituximab along with methotrexate, but this failed to control symptoms. During this period, she had multiple intramuscular and intravenous methylprednisolone infusions to alleviate the symptoms. In 2020, due to high disease activity (DAS-6.93) and lower infection risk, abatacept was administered for 12 months but had partial response.

Following year, she presented with abdominal pain, deranged liver functions and back pain to hospital with raised inflammatory markers. MRCP suggested biliary ductal dilatation secondary to distal biliary stricture and was managed conservatively. MRI of the whole spine confirmed inflammatory changes within the spinal column mainly involving lumbar and sternoclavicular joints, consistent with inflammatory arthropathy. HLA B 27 was negative.

Her disease continued to progress (DAS28 7.7, CRP 76mg/L). Her mobility status reduced drastically by this point leading to bed sores with risk of infection. Hence switched to sarilumab with weekly methotrexate and tapering steroids. Unfortunately, 6 months later she developed pneumonia requiring hospitalization and she refused to go back on sarilumab.

Since early 2023 she has been on tocilizumab, methotrexate and oral prednisolone. Tocilizumab was given fortnightly as she developed neutropenia and haematology input was taken. In the last few months, she developed red, gritty eyes and was diagnosed with scleritis. Currently on steroid eye drops with advice on further immunosuppression.

**Discussion:**

This case focuses on the multiple challenges in managing treatment resistant rheumatoid arthritis.

Even with the availability of a wide range of biologics to manage RA, the patient continues to have SOB, pressure sores, active synovitis, inflammatory back pain with lumbar involvement, scleritis and neutropenia.

There are times when it is difficult to achieve remission despite treatment and that is referred to as resistant or difficult-to-treat rheumatoid arthritis (D2T RA). The above case highlights one such scenario where resistant RA was further complicated with multi system involvement. Patients with interstitial lung disease are at high risk of developing pneumonia and immunosuppression increases that risk further. This can be seen in our case where she required hospitalisation with temporary suspension of her immunosuppressant leading to further worsening of the disease activity.

Along with peripheral joints, spine involvement led to significant reduction in her mobility status resulting in development of bed sores. There is literature extensively discussing the impact of rheumatoid arthritis (RA) on the cervical spine. However, there has been limited focus on the rarer occurrence of rheumatoid involvement in the thoracic and lumbar regions.

In addition, there was patient’s altered perception of rheumatoid arthritis and its symptoms, influencing the medication adherence and coping mechanism, which added to the management dilemmas.

Moreover, one cannot escape from the impact of COVID pandemic and isolation measure that may have created more anxiety among our patients.

Overall, now patient continues to be with high disease activity, infection risk, reduced mobility, multi-biologic resistance in the background of neutropenia which has posed real challenge in her management. Patient specific biologic treatment would help in such situation, and we need more evidence to introduce this in clinical practice.

**Key learning points:**

Cervical spine involvement is well known among patients with RA but it is important to be aware that other areas of the axial skeleton may also be affected. Recognizing these symptoms and initiating early investigations are crucial steps towards prompt diagnosis and appropriate management, ultimately leading to improved outcomes for individuals with RA.

There is a need for more evidence in developing a patient specific treatment for patients with difficult-to-treat rheumatoid arthritis (RA) who are unable to attain the treatment goal with current biologics strategies.

Patients perception, belief about the disease and adherence to medication influence the disease management and thus clinicians need to prioritize patient education about the disease to enhance the effectiveness of treatment early in the treatment.

